# Rare Co-Existent Dermatitis Herpetiformis and Psoriasis in a Child: A Causal Relationship?

**DOI:** 10.7759/cureus.29218

**Published:** 2022-09-15

**Authors:** Ajeet Singh, Satyaki Ganguly, Namrata Chhabra, Vandita Singh

**Affiliations:** 1 Dermatology, All India Institute of Medical Sciences, Raipur, IND; 2 Pathology and Laboratory Medicine, All India Institute of Medical Sciences, Raipur, IND

**Keywords:** gluten sensitivity, immunofluorescence, immunology, psoriasis, dermatitis herpetiformis

## Abstract

Dermatitis herpetiformis (DH) is an auto-inflammatory skin disease that is linked to gluten sensitivity and is related to celiac disease (CD). Psoriasis is an inflammatory skin disorder found to have an association with the celiac disease, according to various genetic and epidemiological studies. We report a 12-year-girl who presented with multiple tense blisters along with red raised, scaly and itchy lesions over her body. She was a known case of psoriasis and was diagnosed as dermatitis herpetiformis in an immunofluorescence study. In this case report, we want to highlight the fact that the co-existence of dermatitis herpetiformis and psoriasis could be more than a mere coincidence. In our patient's previously uncontrolled psoriasis and dermatitis herpetiformis both improved after a gluten-free diet along with systemic therapy.

## Introduction

Dermatitis herpetiformis (DH) is an auto-inflammatory cutaneous disease that is linked to gluten sensitivity and is considered the specific cutaneous manifestation of celiac disease (CD) [1]. Psoriasis has been found to have an association with the celiac disease according to various genetic and epidemiologic data [2,3]. The onset of DH is usually in the fourth and fifth decades, although individuals of any age can be affected [1]. Here, we report a rare case of dermatitis herpetiformis in a child who was a known case of chronic plaque psoriasis.

## Case presentation

A 12-year-old female child, a biopsy-proven case of chronic plaque psoriasis, presented with multiple vesicles and blisters along with intense itching for the last two months. The patient was diagnosed with psoriasis 1.5 years back, and five months ago, oral methotrexate 5 mg weekly was started, in view of extensive involvement of psoriasis but without much improvement. She has developed tense blisters at the site of itching involving the chest, back, and both extremities. There was no other significant history.

**Figure 1:**
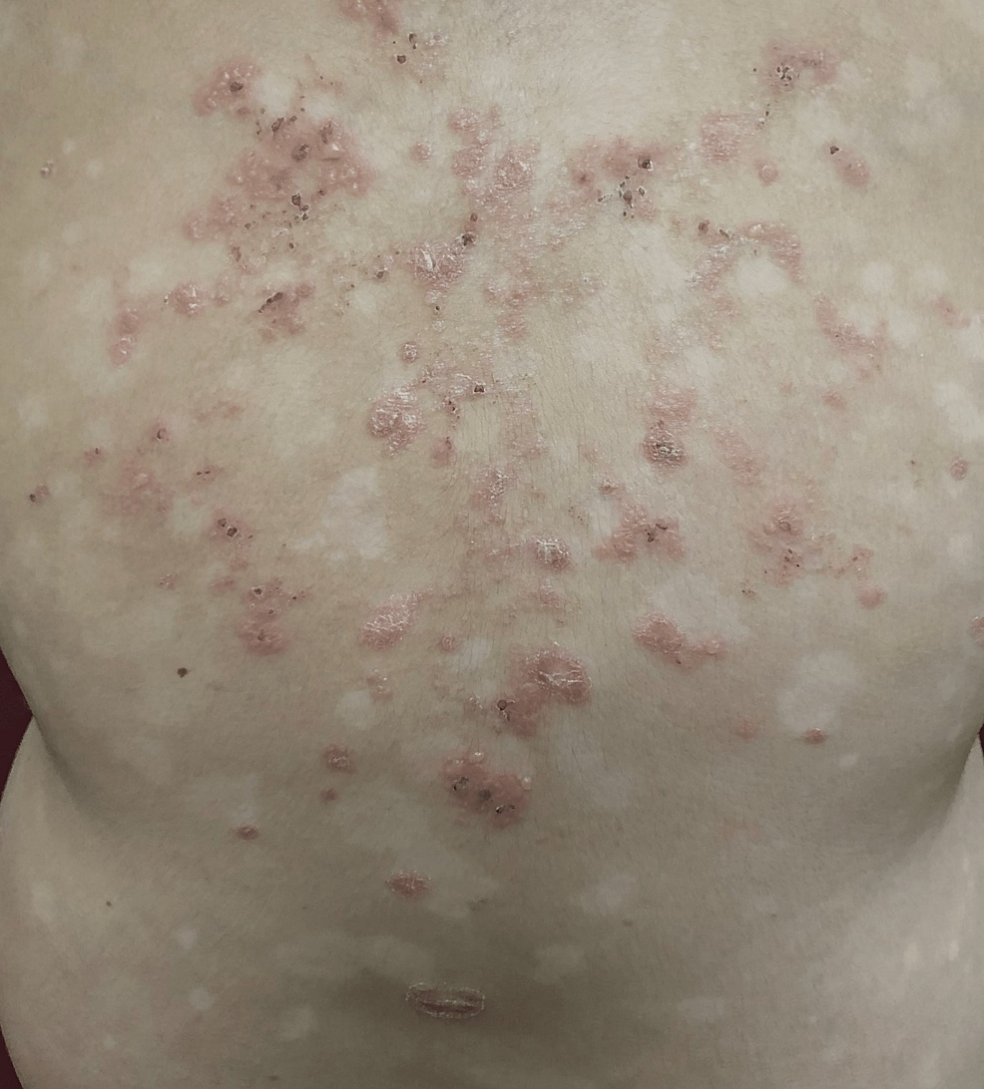
Multiple, clear fluid-filled blisters present over the erythematous, indurated, scaly plaques over the back

**Figure 2:**
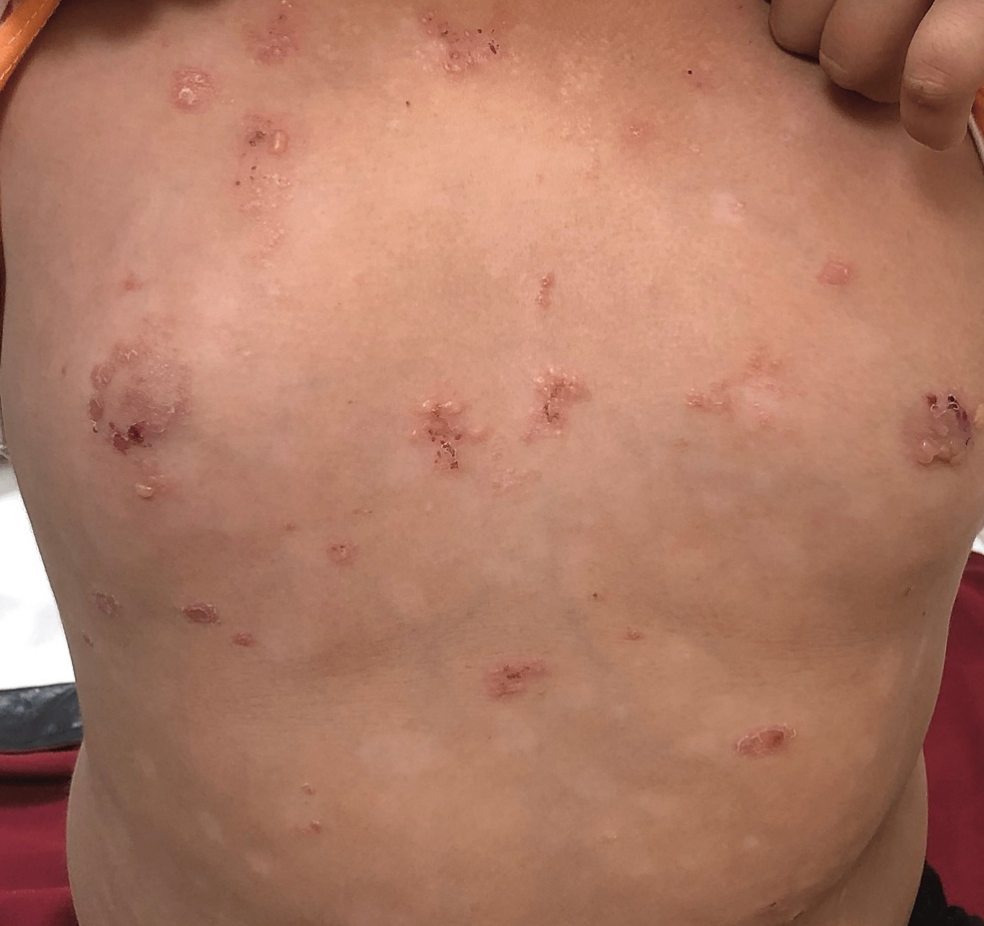
Blisters present on the periphery of the psoriatic plaques as well as on the normal skin over the chest region

Cutaneous examination revealed well-defined, symmetrically distributed small, erythematous, indurated plaques covered with semi-adherent, dry, micaceous scales present over the back, chest, and extensor aspect of upper and lower extremities and face. Multiple clear fluid-filled, tense vesicles and bullae on an erythematous base were present on the periphery of indurated psoriatic plaques, as well as normal skin (Figures 1, 2).

**Figure 3:**
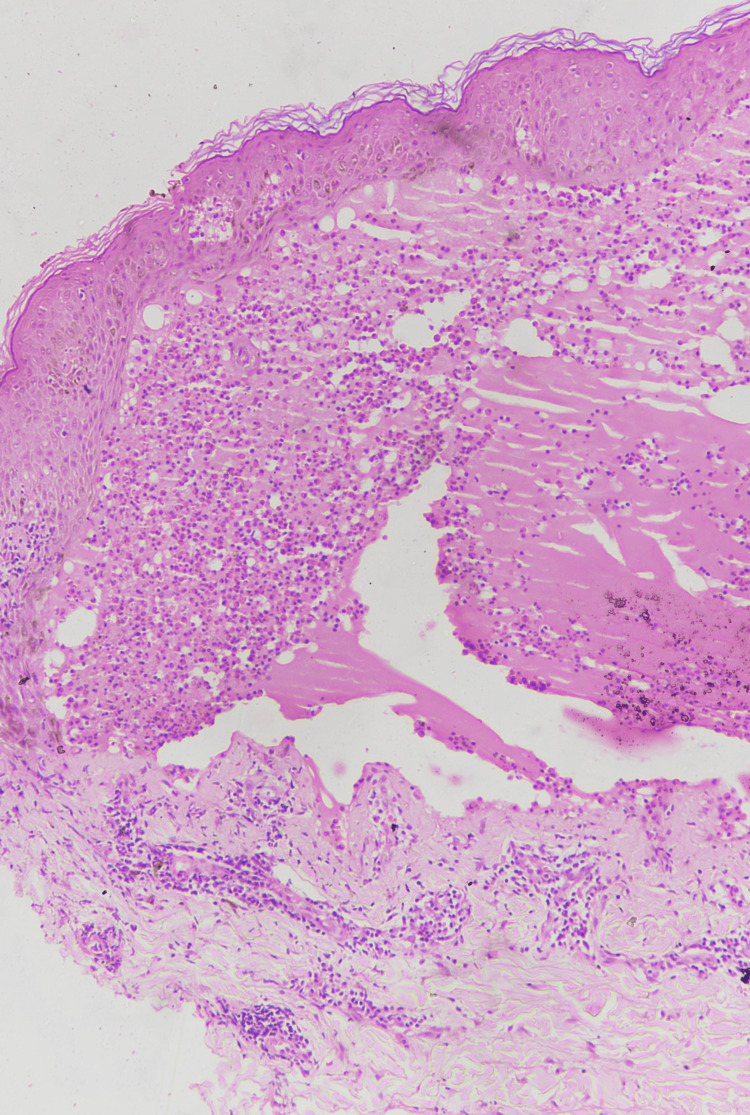
Hematoxylin and Eosin section showing subepidermal blister with predominantly neutrophilic infiltrate and perivascular lymphocytic and neutrophilic infiltrate in the dermis. (10×)

Oral, genital, and ocular mucosa and scalp were spared. Histopathological examination of intact blister showed a subepidermal bulla containing mainly neutrophils and eosinophils, and the dermis showed perivascular and periadnexal lymphohistiocytic and neutrophilic infiltrate, more concentrated in dermal papillae (Figure 3).

**Figure 4:**
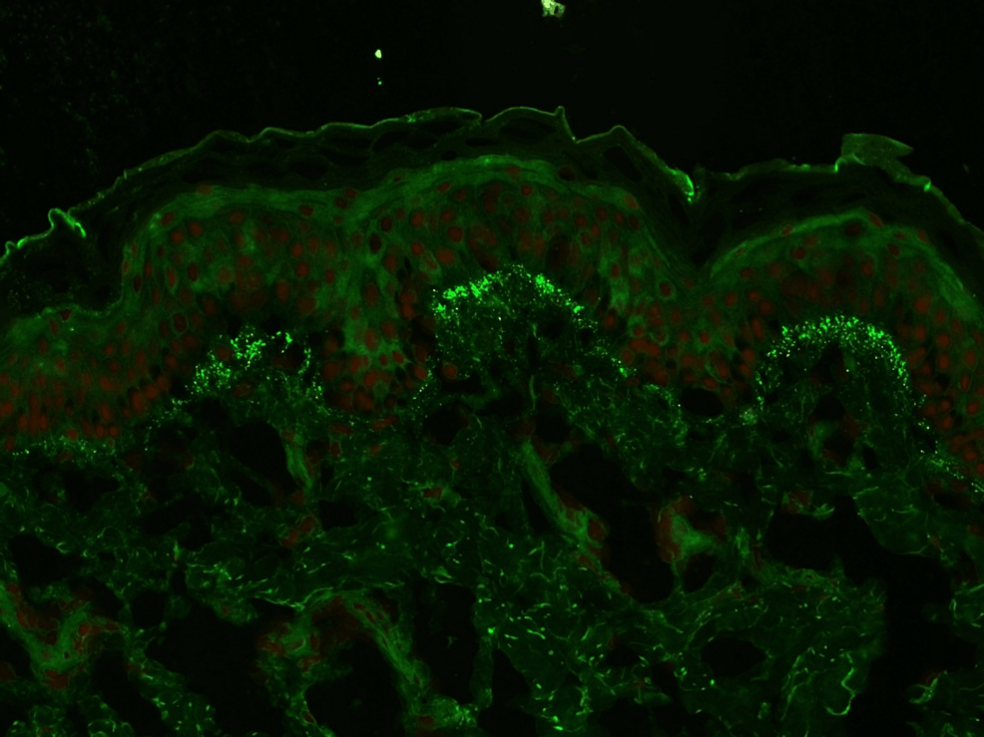
Direct immunofluorescence showing papillary deposits of Ig A antibodies

Direct immunofluorescence (DIF) showed granular staining of the basement membrane zone with IgA only with more intensity over dermal papillary tips (Figure 4).

**Figure 5:**
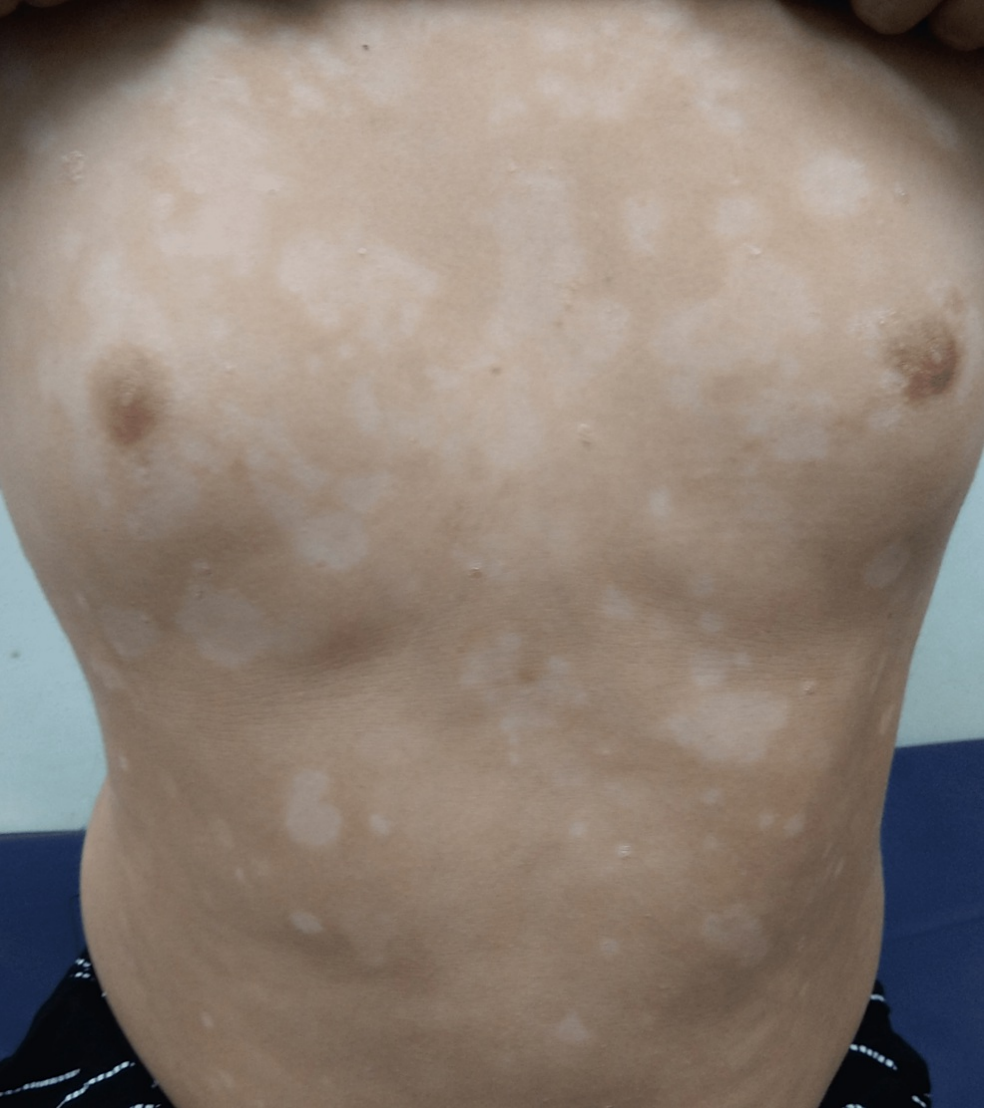
Multiple, hypopigmented macules present over the chest and trunk reminiscent of healed psoriatic plaques

Hence, a diagnosis of dermatitis herpetiformis and chronic plaque psoriasis was made. Her routine investigations, serum tissue transglutaminase (tTG), and gliadin antibodies were within normal limits. She was started on methotrexate 7.5 mg once a week, along with folic acid and dapsone 50 mg per day, and a strict gluten-free diet. Follow-up after 15 days showed most of the DH lesions resolved along with control in itching while psoriatic lesions were persisting. On the next follow-up after six weeks, the patient had no active psoriatic or DH lesions (Figure 5).

The patient is currently on dapsone 25 mg per day and methotrexate 5 mg once per week with well-controlled psoriasis and DH.

## Discussion

Psoriasis is one of the most common immune‑mediated skin disorders with a prevalence of approx. 2%-3% in the adult population, whereas DH is a rare disease, occurring predominantly in Caucasian individuals with prevalence ranging between 1.2 to 39.2 per 100.000 in the USA [2,3]. According to various genetic and epidemiologic studies, there is a definite association between psoriasis and celiac disease with the presence of anti-gliadin antibodies and improvement of psoriasis after starting the gluten-free diet in patients with celiac disease [4,5]. On the other hand, DH is a specific cutaneous manifestation of celiac disease (CD) that is linked to gluten sensitivity. Both conditions are mediated by immunoglobulin (Ig) A, and the diagnosis of DH depends on the detection of granular deposits of IgA within the skin [1].

Our extensive review of literature has revealed only a few case reports of co-existent dermatitis herpetiformis and psoriasis, with the latest report from Australia in 2021 [6-8]. A cross-sectional study by Woo et al. [4] has reported the presence of IgG and IgA anti-gliadin antibodies (AGA), IgA tTG antibodies, and IgA anti-endomysial antibodies (EMA) in more than 16% of patients with psoriasis and correlated with severe psoriatic disease activity. Maki [9] and Nagui et al. [10] also found a similar association and suggested that this might be because abnormal intestinal mucosal pathology or gluten sensitivity in patients with psoriasis may start early in life. The association between CD and psoriasis could be because of the excessive production of Interleukin (IL) -1 and IL‑8 by hyperproliferating keratinocytes in psoriasis, thereby causing mucosal inflammation in response to dietary gluten [11].

The pathogenesis of DH involves a complex interplay between autoimmune factors, such as HLA predisposition, genetics, and environment. Both gluten sensitivity and DH have a common genetic basis [12]. A hypothesis regarding the pathogenesis of DH states that keratinocytes would release eTG into the bloodstream, where it would form immune complexes with IgA, which would then deposit in the dermal papillae. A neutrophilic inflammatory infiltrate is found in skin lesions of DH patients, usually in the papillary dermis, the same site of IgA deposition. The formation of vesiculobullous lesions is derived from collagenase and elastase production by neutrophils, which leads to basement membrane destruction [13].

Patients with DH have increased levels of IL-8, which is one of the initiators of the neutrophilic influx in the dermis, and keratinocytes in psoriasis could be the trigger for increased IL-8 secretion. Further, immuno-histochemical analysis of skin biopsies and measurement of serum concentrations have identified enhanced expression of IL-17 in patients with DH, which is an integral inflammatory mediator in the pathogenesis of psoriasis [14]. According to a study by Cianci et al., [15] increased frequency of the Ig heavy-chain HS1,2-A enhancer*2 allele was seen in patients with DH, plaque psoriasis, and psoriatic arthritis, suggesting a differential immune response induction involving IgA dysregulation in both these diseases.

The patient in the present case had tense bullae present mainly over psoriatic plaques, an unusual presentation suggesting koebnerization of the DH lesions as a result of the epidermal inflammation yielded by psoriasis. In our case, there was a remarkable improvement in both psoriatic as well as DH lesions after administering dapsone, methotrexate, and a strict gluten-free diet, while in the case reported by Lee et al., [8] the patient did not have any significant improvement in psoriasis after gluten-free diet but responded to secukinumab. Other studies have also demonstrated improvements in psoriatic lesions in patients who followed a gluten-free diet with no additional pharmacologic treatment [16].

## Conclusions

Our case helps to take forward the notion that the association between psoriasis and DH/CD could be at a pathophysiological level rather than a coincidental finding, and serological analysis to rule out undiagnosed celiac disease should be performed more frequently in psoriatic patients to improve the disease prognosis in psoriasis. Further, as our patient had only DH but not CD, could it be that even apart from CD, psoriasis has an independent association with DH, and possibly we need to do DIF in psoriasis patients with severe pruritus and excoriated lesions?
